# Supplementary material for: Antiviral Functions of Type I and Type III Interferons in the Olfactory Epithelium
Source: Biomolecules. 2023 Dec 8;13(12):1762. doi: 10.3390/biom13121762 (PMC10741941; doi:10.3390/biom13121762)
Supplement: Supplementary file 1 [file biomolecules-13-01762-s001.zip › biomolecules-2722114-supplementary.pdf]

Source File for Figure 5

Figure 5A

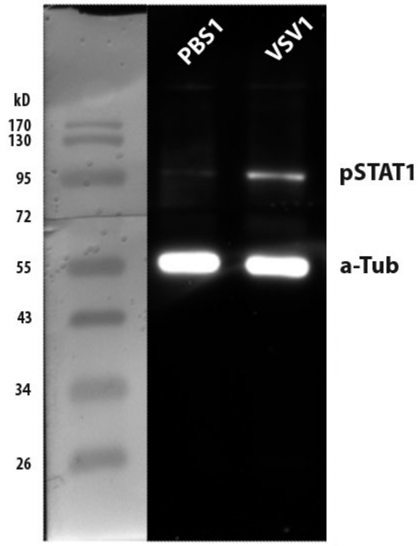

Figure 5A Full length western blot for pStat1. Both pStat1 and a-Tub proteins were detected simultaneously on the same membrane. pStat1 signals shows at ~100 kD and a-Tub signals at 55 kD. The blot contains two lanes, PBS OE lysate on the left and VSV infected OE lysate on the right lane.

Figure 5B

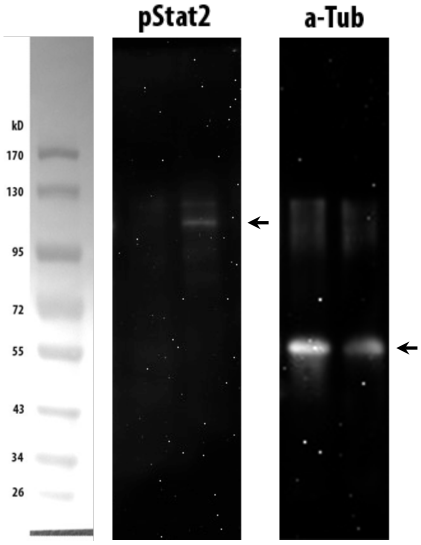

Figure 5B Full length western blot for pStat2. The blot on the left was probed for pStat2. The blot on the right was the same membrane used with the pStat2 signals stripped and re-probed for a-Tub. Arrows point to the pStat2 and a-Tub band respectively. The blot contains two lanes, PBS OE lysate on the left and VSV infected OE lysate on the right lane.
